# Cutaneous Collateral Axonal Sprouting Re-Innervates the Skin Component and Restores Sensation of Denervated Swine Osteomyocutaneous Alloflaps

**DOI:** 10.1371/journal.pone.0077646

**Published:** 2013-10-18

**Authors:** Zuhaib Ibrahim, Gigi Ebenezer, Joani M. Christensen, Karim A. Sarhane, Peter Hauer, Damon S. Cooney, Justin M. Sacks, Stefan Schneeberger, W. P. Andrew Lee, Michael Polydefkis, Gerald Brandacher

**Affiliations:** 1 Department of Plastic and Reconstructive Surgery, Johns Hopkins University School of Medicine, Baltimore, Maryland, United States of America; 2 Department of Neurology and Neurosciences, Johns Hopkins University School of Medicine, Baltimore, Maryland, United States of America; 3 Department of Visceral, Transplant and Thoracic Surgery, Center of Operative Medicine, Innsbruck Medical University, Innsbruck, Austria; di Pompeo d'Illasi, University of Rome, Italy

## Abstract

Reconstructive transplantation such as extremity and face transplantation is a viable treatment option for select patients with devastating tissue loss. Sensorimotor recovery is a critical determinant of overall success of such transplants. Although motor function recovery has been extensively studied, mechanisms of sensory re-innervation are not well established. Recent clinical reports of face transplants confirm progressive sensory improvement even in cases where optimal repair of sensory nerves was not achieved. Two forms of sensory nerve regeneration are known. In regenerative sprouting, axonal outgrowth occurs from the transected nerve stump while in collateral sprouting, reinnervation of denervated tissue occurs through growth of uninjured axons into the denervated tissue. The latter mechanism may be more important in settings where transected sensory nerves cannot be re-apposed. In this study, denervated osteomyocutaneous alloflaps (hind- limb transplants) from Major Histocompatibility Complex (MHC)-defined MGH miniature swine were performed to specifically evaluate collateral axonal sprouting for cutaneous sensory re-innervation. The skin component of the flap was externalized and serial skin sections extending from native skin to the grafted flap were biopsied. In order to visualize regenerating axonal structures in the dermis and epidermis, 50um frozen sections were immunostained against axonal and Schwann cell markers. In all alloflaps, collateral axonal sprouts from adjacent recipient skin extended into the denervated skin component along the dermal-epidermal junction from the periphery towards the center. On day 100 post-transplant, regenerating sprouts reached 0.5 cm into the flap centripetally. Eight months following transplant, epidermal fibers were visualized 1.5 cm from the margin (rate of regeneration 0.06 mm per day). All animals had pinprick sensation in the periphery of the transplanted skin within 3 months post-transplant. Restoration of sensory input through collateral axonal sprouting can revive interaction with the environment; restore defense mechanisms and aid in cortical re-integration of vascularized composite allografts.

## Introduction

Vascularized Composite Allotransplantation (VCA) is an innovative reconstructive strategy for optimal restoration of appearance, anatomy and function following devastating tissue loss [1]. Widespread clinical application of VCA, however, can only be realized if functional recovery is maximized and immunosuppression is minimized [2]. In recent years, new treatment options to improve and enhance motor nerve regeneration after VCA have been an increasing focus of research [3]. Mechanisms of sensory return, however, are still ill defined because standardized assessments of sensory recovery in animal models are not well established. 

Available clinical reports from full and partial-face transplants describe progressive but variable sensory improvement even without optimal sensory nerve repair [4]. Several explanatory mechanistic hypotheses have been postulated, including regeneration from the recipient bed (regenerative nerve sprouting) or alloflap margins (collateral nerve fiber sprouting) and transmission of sensory inputs through afferent facial nerve fibers [4]. Objective evidence regarding the independent contributions of these factors, however, is lacking. The neurotrophic requirements and timeframes of the two forms of sensory re-innervation (regenerative and collateral sprouting) are very different and likely would require different strategies to be targeted therapeutically [5–7]. Ultimately, optimal functional recovery will require potentiation of each of these forms of nerve regeneration. 

Human skin is densely innervated with nerve bundles that typically course through the dermis and form horizontal sub-epidermal neural plexuses in the papillary dermis. From the papillary dermal plexuses these nerves grow vertically, lose their Schwann cell investment at the dermo-epidermal junction, penetrate the epidermal basement membrane, ascend between the keratinocytes, and terminate in the keratin or superficial layers of the stratum spinosum as free nerve endings. Hence, cutaneous innervation consists mainly of unmyelinated fibers, which account for approximately 90% of all dermal nerve fibers [8]. Since conventional electrodiagnostic methods are not useful in assessing these unmyelinated cutaneous nerve fibers simply because this population is "invisible" to nerve conduction velocity studies, our group has validated the well-established epidermal nerve fiber density test as a tool to measure regenerating fibers in different sensory neuropathies ([9–13] (Table 1). 

**Table 1:** Experimental findings from human studies utilizing intracutaneous axotomy model.

**Study**	**Year**	**Type of subjects**	**Site of axotomy**	**Follow up duration**	**Sprouting Rate**
Rajan et al.	2003	Healthy Subjects (n=9)	Back	23 months	5-20 μm/day
Hahn et al.	2006	Healthy subjects treated with timcodar dimesylate or placebo (n=52)	Distal thigh	56 days	8.5 μm/day (No difference between treatment and placebo groups)
Hahn et al.	2007	Healthy subjects (n=5) HIV+ subjects (n=5)	Distal Thigh	60 days	Healthy subjects 9.78 μm/day HIV+ Subjects 5.43 μm/day
Ebenezer et al.	2011	Healthy subjects (n=10) Diabetic subjects (n=10)	Distal Thigh	2-3 months	Healthy subjects 20 μm/day Diabetic Subjects 10 μm/day

Slow and steady axonal regeneration and collateral sprouting was significantly reduced in diabetic patients and Human Immunodeficiency Virus positive individuals when compared to healthy controls.

We herein conducted the first investigational large animal study exploring the role of such collateral sensory axon sprouting in the setting of VCA employing a unique immunohistochemical analysis technique. 

## Materials and Methods

### Animals

Osteomyocutaneous flap transfer (heterotopic hind limb transplant) was performed between MHC-defined MGH miniature swine (Massachusetts General Hospital, Boston MA). A total of twelve animals were included in the study: no treatment control (n=3), tacrolimus only control (n=3), tacrolimus combined with co-stimulatory blockade, CTLA4Ig (n=3) and tacrolimus combined with co-stimulatory blockade, CTLA4Ig and donor bone marrow (BM) infusion (n=3). Prolonged rejection free graft survival (>150 days) was achieved in animals that received combined costimulatory blockade and BM infusion. Collateral cutaneous axonal sprouting was assessed in one of the long-term survivors demonstrating robust immune tolerance with no clinical or histologic evidence of rejection.

This study was carried out after approval from Johns Hopkins University Animal Care and Use Committee guidelines (Protocol SW11M64) and in strict accordance with the recommendations in the Guide for the Care and Use of Laboratory Animals of the National Institutes of Health. 

### Surgical Procedure

**Figure 1:**
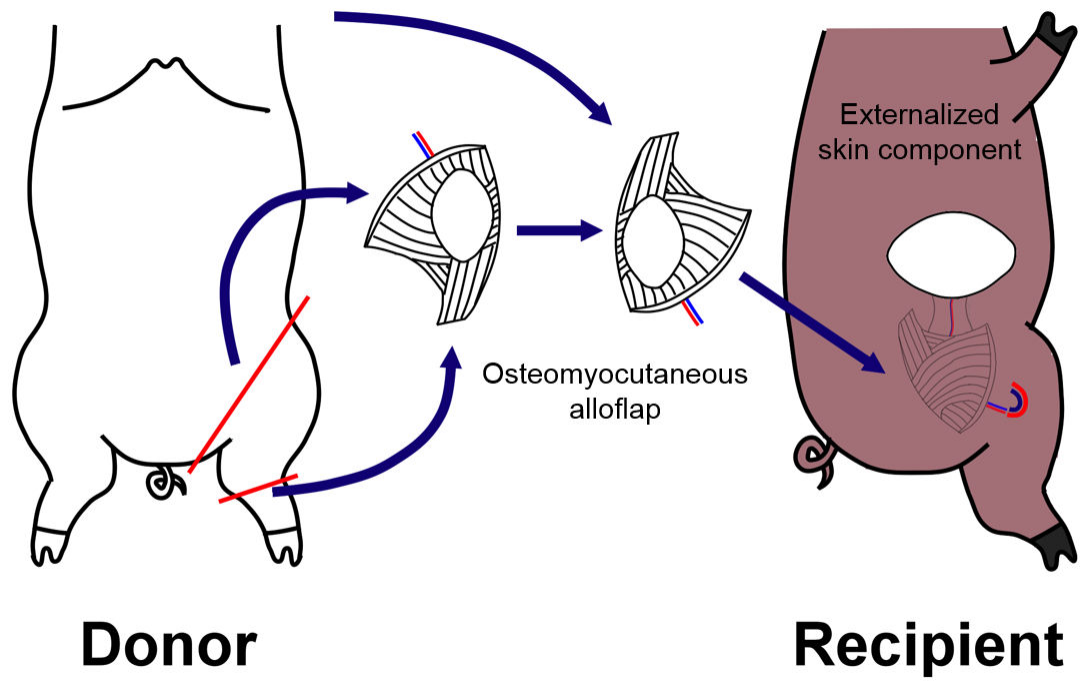
Schematic diagram of swine heterotopic hind limb transplant. Osteomyocutaneous flap consisting of femur, tibia, fibula, knee joint, overlying muscles and skin is transplanted to a subcutaneous pocket in recipient with externalized skin component.

The alloflap consisted of the distal femur, knee joint, proximal tibia and fibula, surrounding muscle and a well-vascularized skin component (average diameter: 7±1 cm) (Figure 1). Near infrared laser angiography was used to identify the vascular perforator in the anteromedial thigh prior to flap harvest and perfusion with Histidine-Tryptophan-Ketoglutarate (HTK) (Custodiol, Newtown PA). The osteomyocutaneous flap was transplanted to a subcutaneous abdominal pocket in the recipient with end-to-end femoral anastomosis *without* neurorrhaphy (Figure 1). Two flaps were harvested from each donor and transplanted to two recipients in a way that the right limb was transplanted to the left groin and vice versa. The skin component of the graft was positioned in a standardized fashion at the dorsolateral abdominal wall equidistant from the spine and ventral midline. 

### Immunotherapy

Immunotherapy consisted of co-stimulation blockade (Orencia, Bristol-Myers Squibb, NY) on postoperative day 0, 2, 4 and 6 and tacrolimus monotherapy (30 days, target level 10-15 ng/ml). Functional and histologic assessment of sensory recovery continued for 8 months post-transplant. 

### Functional Analysis

Sequential pinprick tests were performed using a sharp Neurotip within a Neuropen (Owen Mumford Inc. GA) to ensure quantifiable force of 40g exerted onto the skin until the guiding markers on the Neuropen were aligned. An area of skin far away from flap was first tested while animal was distracted using a food incentive. The periphery of flap (1 cm from the junction with native skin) as well as center of the graft was then evaluated in a similar manner. Testing of different regions was performed with a time gap of at least one hour to prevent habituation or sensitization. 

### Immunohistochemical Analyses

Objective histologic assessment of cutaneous nerve innervation was performed on skin rolls excised at 0.5cm intervals from the periphery (margin of the graft anastomosis to the graft center) at the time of euthanasia. Additional skin excisions were performed with a distance of 0.5 cm to the margin at early post-operative days (day 75 and day 100 to confirm denervation and re-innervation). Skin sections were preserved in Zambonis fixative; cryoprotected and sectioned at 50 micrometer intervals. Sections were incubated overnight at 4°C with primary antibodies PGP 9.5 (AbD SeroTec, Raleigh, NC, USA, dilution 1:10,000 in triton buffer), mouse anti-p75 nerve growth factor receptor (Millipore, Temecula, CA, USA, dilution 1:1000 in triton buffer) mouse anti-growth associated protein (GAP)-43/B-50 (marker for regenerating fibers; Millipore, Billerica, MA, 1:500), then with biotinylated secondary antibody and Avidin-Biotin Complex solutions (Vector Labs) for 1hr respectively, followed by substrate incubation for 8-10 min (Vector Laboratories, Burlingame, CA), and counterstaining with Eosin (1%) and Mayer's haematoxylin as described previously (Holland, Annals Neurology). Preparations were viewed with Olympus BH2 and Zeiss Axioscop microscopes. Schwann cell bands within the dermis were identified in the skin sections by the presence of brown colored cytoplasmic staining by p75. 

### Measurement of collateral axonal sprouts

The collateral axonal sprouting distance was defined as centripetal distance along the epidermal basement membrane measured from the junction of native and transplanted skin (measured with Bioquant software; R&M Biometrics) as has been reported previously [12,14]. Collateral sprouting distance divided by number of days yielded the growth rate (mm/day) [11].

## Results

**Figure 2:**
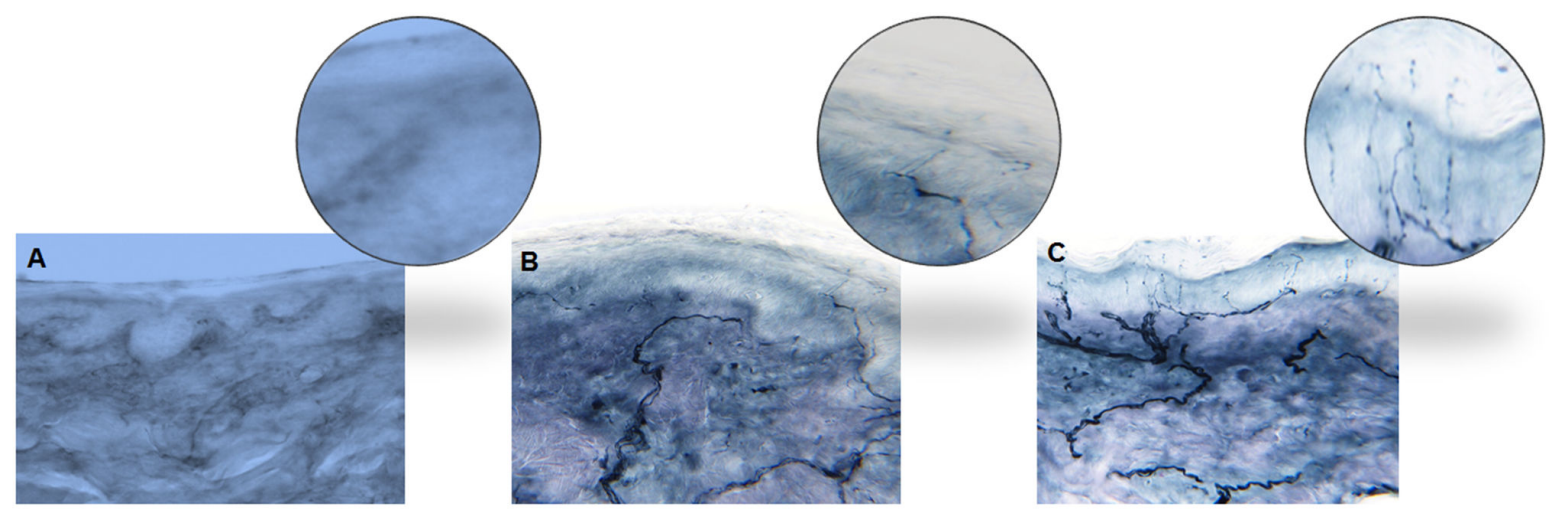
Immunohistochemical staining at Day 75 and 100. (A) Lack of PGP9.5 staining at Day 75 demonstrating complete denervation. (B) Axonal sprouts visualized in a 3 mm punch biopsy 0.5 cm from skin margin at Day 100. (C) Native skin adjacent to flap demonstrating dense intra-epidermal axons.

**Figure 3:**
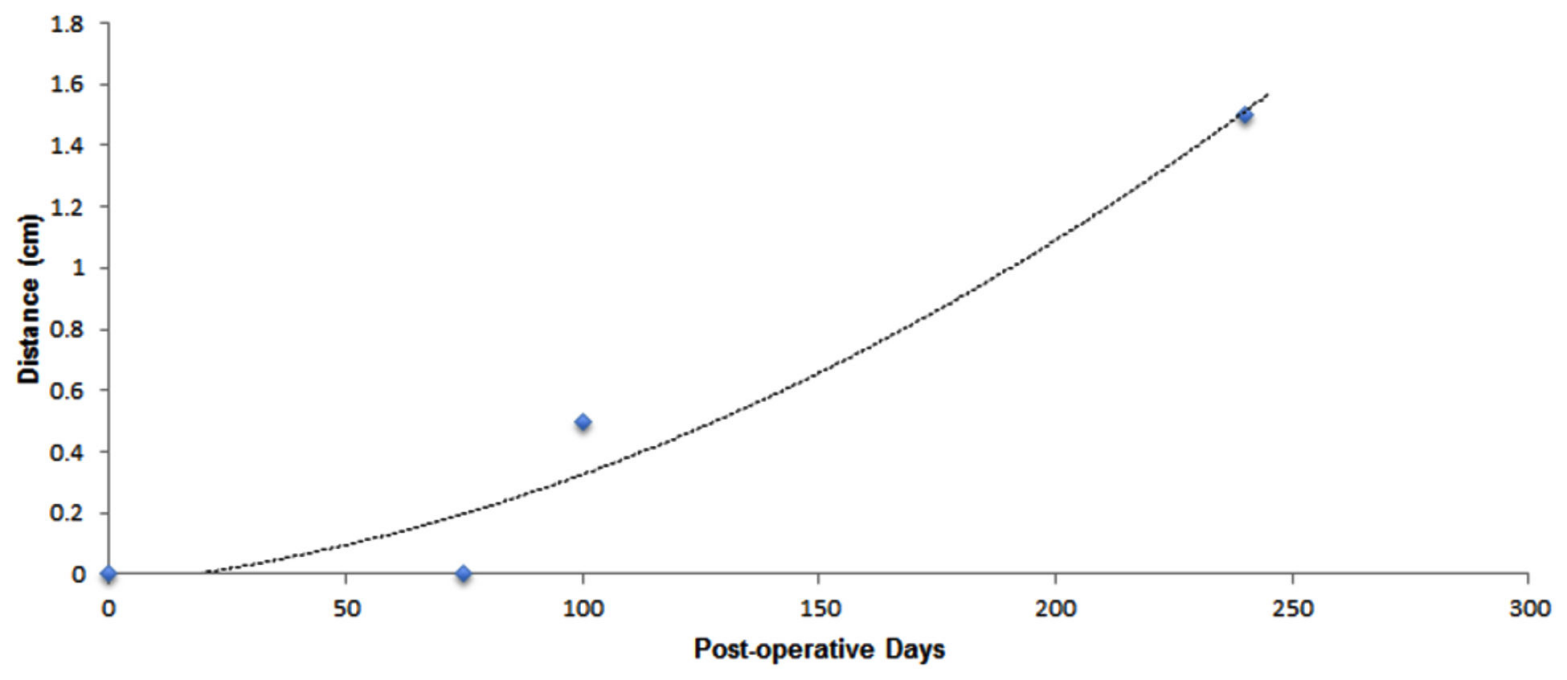
Collateral sprouting rate. Polynomial regression model demonstrating centripetal distance of collateral axonal sprouting from adjacent native skin as a factor of time since transplant with estimated rate of regeneration 0.06 mm per day.

**Figure 4:**
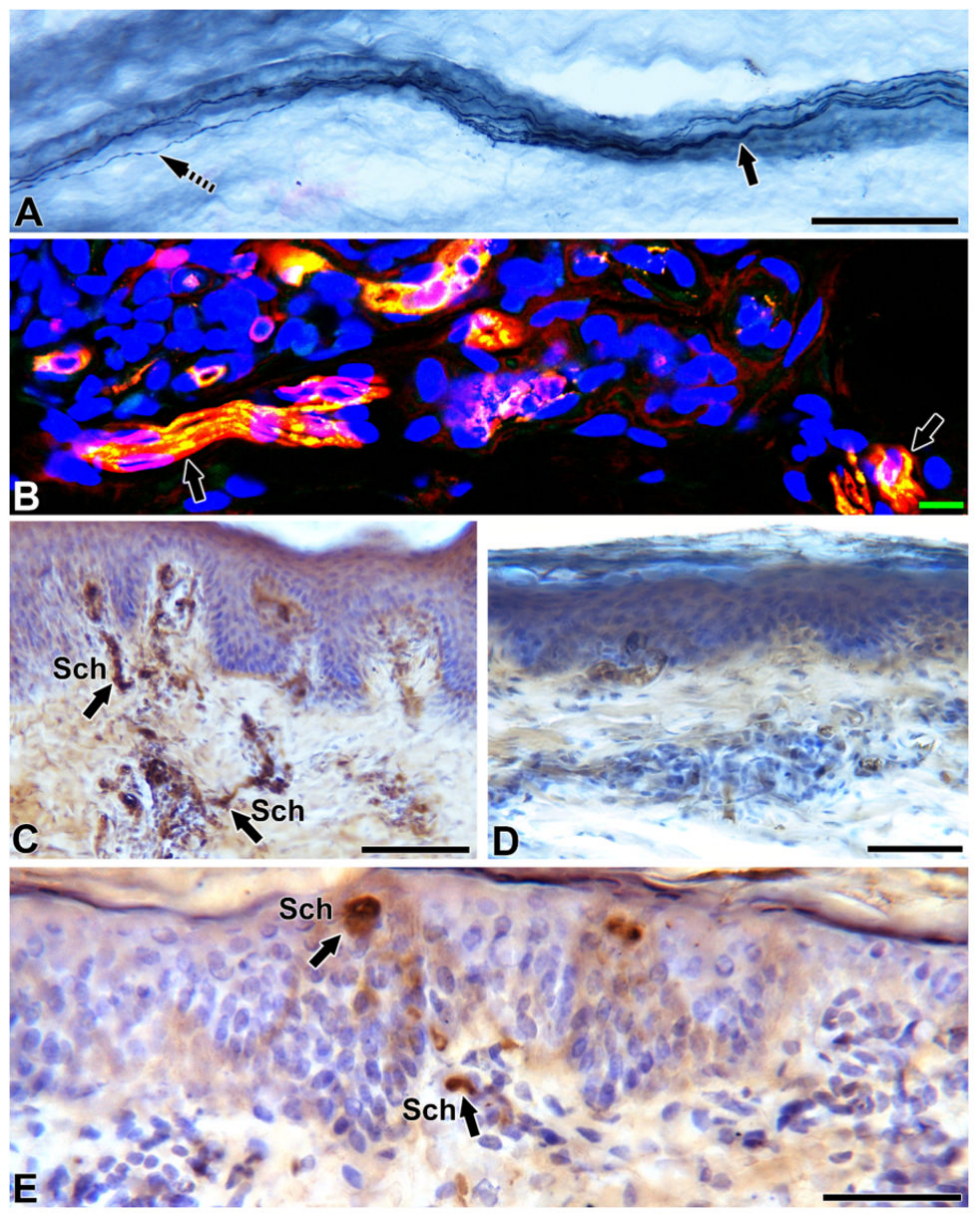
Schwann cell staining (p75) and confocal microscopy triple staining with PGP9.5, GAP43 and DraQ5 at the junction between native skin and alloflap. Skin sections immunostained with PGP9.5 (A), nerve growth factor receptor, p75 (C, D and E) and confocal microscopy montage triple-stained with the axonal marker PGP9.5 (red), GAP 43 (green) indicating co-localization as yellow and the nuclear marker DraQ 5 (blue). (A) At the junction between native and grafted skin, a thick deep dermal nerve bundle ( arrow) surrounded by dense collagen extending out collateral sprouting fibers ( slashed arrow) towards the grafted skin. (B) These dermal bundles at the grafted skin exhibited newly regenerating fibers (yellow, arrow). (C) Native skin served as control showing thick epidermal layers and vertical Schwann cell bands (arrows, Sch) entering from the deeper dermis into the papillary dermis. (D) On day 240, Schwann cells in the center of grafted flap had completely degenerated (lack of p75 staining) indicating that collateral sprouting only could serve as the major pathway for re-innervation. The epidermis is thin at this site. (E) Schwann cell tubes at the papillary dermis (arrows, Sch) at the junction between native and the donor flap indicate the Schwann cell support for the collateral sprout guidance. The border shows dense aggregation of inflammatory cells (broken arrow). Scale bar: A=100µm, B=10µm, C and D=50 µm.

**Figure 5:**
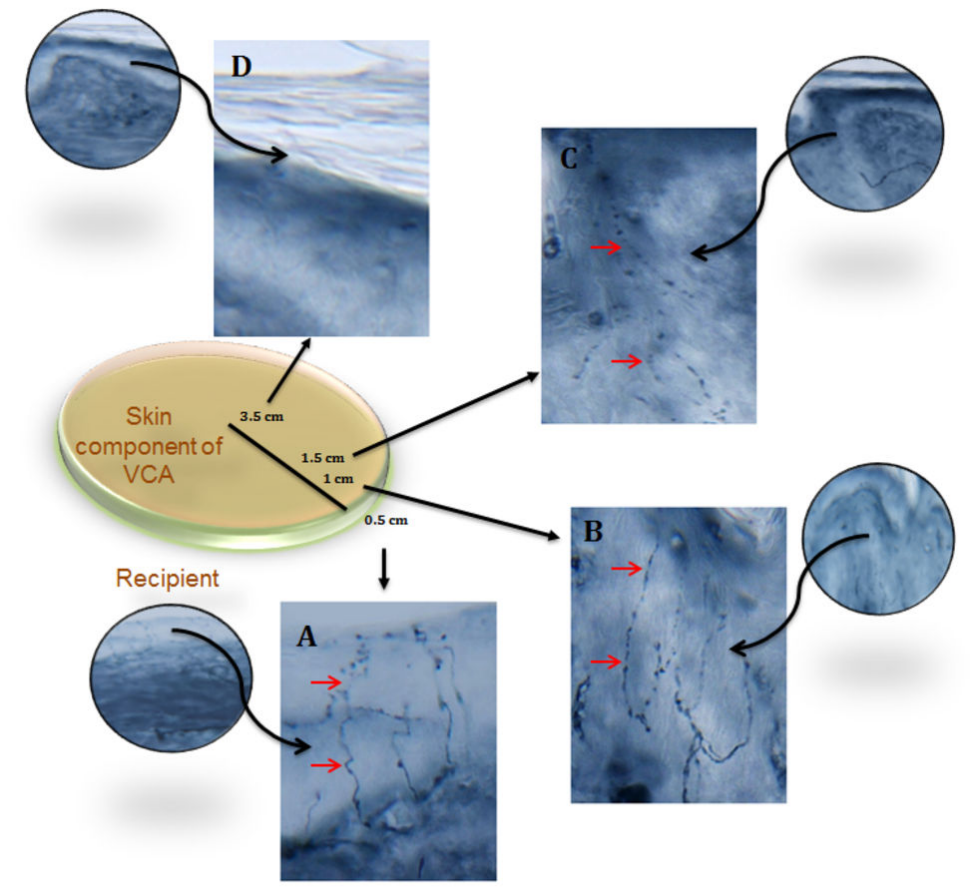
Sequential sections of skin component of allograft (Day 240 post-transplant). PGP9.5 (pan-axonal marker) demonstrates dense epidermal fibers (solid arrow) in native skin 0.5 cm away from the graft (A). Intra-epidermal fibers also visualized in skin component of VCA at 1cm (B) and 1.5 cm (C) away from the junction with native skin. No epidermal fibers seen in the center of alloflap (D).

All osteomyocutaneous alloflaps were well perfused and viable. The immunosuppression protocol was well tolerated. Three of five animals had indefinite rejection-free survival of the skin component, permitting long-term functional and histologic analysis. As expected, there was complete denervation of the transplanted skin post-operatively, as determined by lack of PGP9.5 staining (Figure 2). This is also consistent with previous human studies of skin innervation following sural nerve biopsy [11]. Collateral axonal sprouts from adjacent recipient skin extended into the denervated graft skin in a stereotypic pattern, along the dermal-epidermal junction towards the center as evidenced by immunohistochemical stain on day 100. Few axons extended vertically into the stratum spinosum as intraepidermal fibers. On day 100 post-transplant, regenerating sprouts reached 0.5 cm into the flap. Eight months post-transplant, both organized dermal and fine epidermal fibers were observed up to 1.5 cm from the margin, corresponding to a rate of regeneration of 0.06 mm per day (Figure 3). Day 240 transplant skin samples revealed collateral sprouts emerging from deep dermal bundles and elongating centripetally into the denervated area (Figure 4) and the deep dermal sprouts exhibited GAP43 stain positivity confirming new regenerating fibers. Clinically all animals had pinprick sensation in the peripheral portion of the transplanted skin within 3 months, indicating a peripheral-to-central pattern of regeneration (Figure 5). No Schwann cell bands were identified in the dermis on day 240 in the central transplanted skin, but short Schwann cell tubes along the papillary dermis close to the junction between recipient and donor skin indicate that the epidermal basement membrane collagen and Schwann cells support and guide the collateral sprout growth (Figure 4).

## Discussion

While recovery of motor function is recognized as a key result in VCA, sensory recovery remains another, less consistently achieved but important goal [3]. Progressive sensory recovery has been described in patients receiving partial- or full-face transplants without neurorrhaphy [4], but the mechanisms of this recovery have not been explored. Using a translational large animal VCA model, we were able to show that collateral axonal sprouting from the junction with the recipient skin significantly contributes to cutaneous re-innervation of the skin component of the alloflap. 

**Figure 6:**
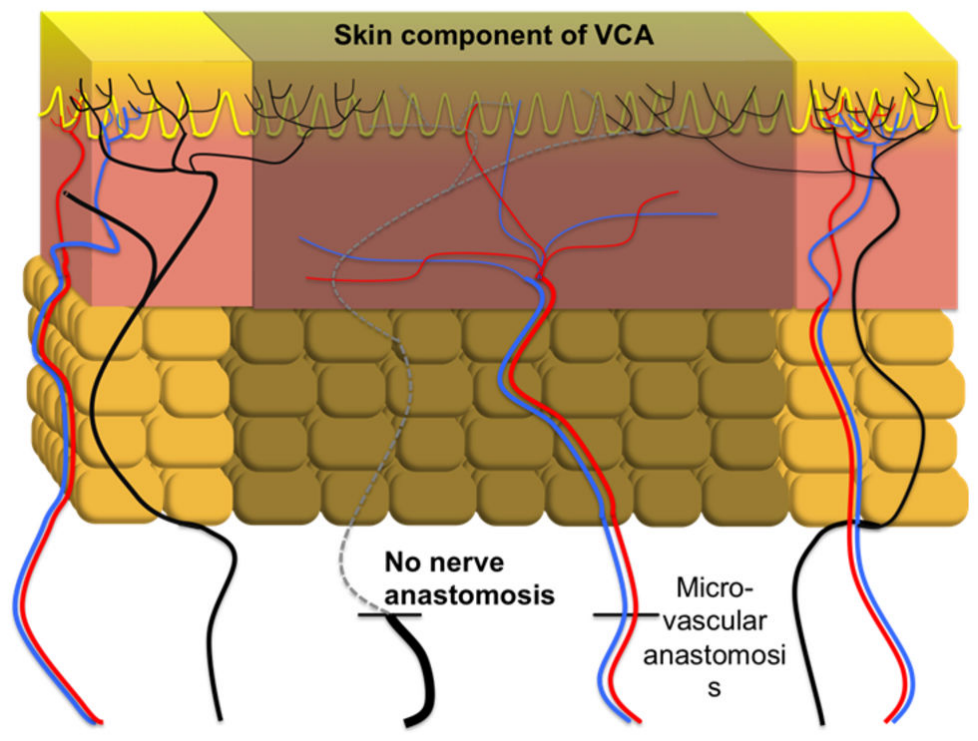
Schematic diagram of a denervated ostemyocutaneous flap. Blood vessels are anastomosed without apposition of nerve endings allowing the regenerative pathway to undergo atrophy (Dotted line). Re-innervation of the denervated VCA epidermis is achieved through collateral sprouting of uninjured axons outside the transplant region (shown in black).

In 2011, Siemionow reviewed sensory outcomes following face transplantation with suboptimal sensory nerve repair with those of conventional nerve repair, face replantation, and free tissue transfer, all of which included a primary nerve anastomosis, using the Medical Research Council Scale as modified by Mackinnon and Dellon[4]. The authors concluded that sensory recovery in such a scenario was comparable to that in the other situations using these clinical outcomes measures, but could only speculate as to the source of the recovery. Based on human experiments of intracutaneous axotomy, two different mechanisms are well known for cutaneous re-innervation following axotomy, regeneration and collateral sprouting. In our large animal investigational study, we isolated the sensory regenerative pathway from collateral axonal sprouting by transplanting a completely denervated composite alloflap (Figure 6). Lack of staining for vertical Schwann cell tubes at day 240 at the center of dermis demonstrates that regenerative sprouting from transected axon stumps is extremely unlikely to occur as the Schwann cell bands through which regenerative axons would travel has been lost. Therefore, we believe that the re-innervation of VCA skin observed is a result of collateral sprouting of uninjured nerve fibers. In order to test this hypothesis we assessed the collateral axonal sprouting using our established immunohistochemical analysis. 

According to the European Federation of Neurological Societies and Peripheral Nerve Society guidelines, immunohistochemical analysis of intra-epidermal nerve fibers is a useful diagnostic tool for objective assessment of peripheral neuropathies [15,16] and has also been compared to a clinical model of intracutaneous axotomy (Table 1) [12,14]. The underlying mechanistic events of Schwann cell changes and axonal degeneration seen in our model are comparable to histologic findings demonstrated by Ebenezer [8,17]. The rate of axonal sprouting in pigs (60 μm/day), however, was relatively faster when compared to our clinical trials with healthy subjects (5-20 μm/day). This can be attributed to species-specific differences as well as the anatomic location of biopsies. In our human trials [8,14], biopsies were obtained from extremities, whereas our large animal model manifests sprouting from abdominal wall, which may contribute to a higher rate of sprouting in addition to species-specific differences. In human face transplantation, sprouting will occur in a centripetal manner owing to the circular shape of flap and can potentially be faster than extremities. One of the limitations of this pilot project is lack of an innervated osteomyocutaneous flap as a control group where regeneration would occur both from regenerative pathway and collateral sprouting. The independent contribution of the two pathways, however, may be difficult to investigate in this large animal model where the limb is being transplanted heterotopically from thigh to trunk. Additional animal models utilizing orthotopic VCA techniques could be useful in analyzing dual methods of sensory re-innervation in the future. 

Based on experience from human studies of incisional axotomy, the rate of collateral sprouting tends to regress as regenerating axons reach the epidermis [13]. Such dual mechanisms of epidermal re-innervation seem plausible in the majority of clinical VCA and ‘innervated’ free flaps. In our denervated flap, collateral sprouting had a linear relationship with time (Figure 3). However, this linear relationship may eventually plateau over time. Even though Rajan [13] demonstrated progressive diminishing of the rate of axonal sprouting in a small series of healthy subjects, the majority of intracutaneous axotomy studies were short duration (2-3 months) and lesions were of smaller size (3 mm). Santanelli et al [18] demonstrated progressive sensory recovery in a denervated deep inferior epigastric perforator (DIEP) flap using sophisticated objective methods and a computer-assisted pressure-specified sensory device. The progressive sensory recovery in our experimental model correlates well with the clinical findings demonstrated by Santanelli et al. However, further experimental studies in VCA models with longer follow-ups are necessary to establish coronal relationships of axonal sprouting. 

In addition to intracutaneous axotomy studies, cutaneous re-innervation following partial and full thickness skin grafts has been assessed. Santoni-Rugiu [19] performed histological analysis on biopsies taken from the center and periphery of skin grafts and flaps in rabbits and found no difference in samples taken from the center and periphery. The first signs of reinnervation were seen at 40 days, and consisted of fibrils in the deeper levels of the grafts. Similarly, Waris [20] demonstrated that three weeks after skin grafting, nerves at the subdermal level under the graft and at the margins were growing toward the denervated area. Bayramiçli [21] demonstrated that skin grafts regain sensory innervation by regeneration of nerve endings in the graft bed, and that innervation is aided by placement of the skin graft over innervated muscle. These grafts, however, differ significantly from the skin component of vascularized flaps and VCA. As opposed to vascularized composite flaps, conventional skin grafts are only a few millimeters thick and can easily re-gain cutaneous innervation through regenerative mechanisms highlighted by Ebenezer [12].

Regeneration across an allogeneic barrier has also been demonstrated in skin grafts. Samulack [22] examined reinnervation of an allogeneic skin transplanted in a non-human primate model and found reinnervation of skin appendages and receptors by host axons, indicating that sensory reinnervation can also occur across Major Histocompatibility Complex (MHC) barriers. This group also found that Merkel cells were lost after transplantation, indicating that mechanisms seen in autografts may not extend to allografts. This held true until Lee demonstrated that an intact vascularized skin in a composite alloflap is immunologically unique when compared to non-vascularized skin grafts and has immune privileged features [23]. Hence the immune mechanisms affecting sensory recovery in skin grafts might not hold true for VCA. 

A number of groups have investigated sensorimotor repair following VCA using animal models. In a rat hemifacial transplant model, Landin compared animals receiving grafts without nerve repairs, and animals undergoing transplant with primary repair of facial nerve branches and the infraorbital branch of the trigeminal nerve [24]. After six weeks, animals undergoing nerve repairs displayed clinical signs of sensory recovery. In contrast, animals that did not undergo nerve repair did not display sensory recovery. Our group [25] found that rats undergoing hemiface transplant with nerve apposition showed return of whisking function 17-20 days following transplant and cortical activity following whisker stimulation at 20 weeks. Animals undergoing transplant without nerve repair did not show return of whisking or electrical activity within the nerves. These findings further potentiate the need for sensory nerve repair in the setting of VCA but could not explain the clinical findings in transplant recipients with sub-optimal nerve repair. Our large animal study as well as extensive human studies on peripheral neuropathies [12,17] demonstrate that collateral axonal sprouting represents an alternative mechanism that can establish sensory re-innervation especially in cases where scarring from trauma and prior surgeries prohibit isolation of sensory nerves.

We utilized tacrolimus in combination with costimulatory blockade as immunomodulation to prolong graft survival. Neuroregenerative effects of tacrolimus have been described in experimental models of regenerative nerve fiber sprouting and have been postulated to play a role in enhanced sensory recovery after VCA. Diamond et al demonstrated a basic biologic difference between axonal sprouting of small sensory nerve fibers and regeneration of axons in sensory nerves: Axonal regeneration after nerve crush is unaffected by anti-neural growth factor (NGF) antibodies and thus appears to occur independently of NGF [26]. In contrast, collateral sprouting is NGF dependent, as shown by increased outgrowth with intradermal injection of NGF [27] and blockade of outgrowth after either local [28] or systemic administration of anti-NGF antibodies [26,27]. These experimental findings are of particular interest in the context of cutaneous axonal sprouting following VCA since tacrolimus has been shown to potentiate NGF-induced neurite outgrowth via the Ras/Raf/MAP kinase pathway [29]. These basic biological differences in regenerative and collateral axonal sprouting may necessitate different therapeutic strategies for enhancing sensory recovery following VCA.

## Conclusion

Collateral axonal sprouting from the periphery extends along the dermal-epidermal junction to provide cutaneous re-innervation to the skin component of reconstructive transplants and free flaps even when sensory nerves are not repaired. Return of sensation through collateral axonal sprouting can revive interaction with the environment and re-establish defense mechanisms, independent of other pathways of sensory re-innervation. Furthermore, basic biological differences in regenerative and collateral axonal sprouting may necessitate different therapeutic strategies for enhancing sensory recovery following VCA. 
